# Transient global amnesia: Linked to a systemic disorder of amino acid catabolism?

**DOI:** 10.1007/s00415-013-6927-x

**Published:** 2013-04-25

**Authors:** Giuseppe Sancesario, Zaira Esposito, Alessia F. Mozzi, Giulia M. Sancesario, Alessandro Martorana, Angela Giordano, Roberto Sorge, Barbara Mari, Gianfranco Spalletta, Maria Grazia Marciani, Sergio Bernardini

**Affiliations:** 1 Department of Systems Medicine, Tor Vergata General Hospital, Faculty of Medicine and Surgery, The University of Rome Tor Vergata, 1 Montpellier Street, 00133 Rome, Italy; 2 Department of Experimental Medicine and Surgery, Tor Vergata General Hospital, Faculty of Medicine and Surgery, The University of Rome Tor Vergata, Rome, Italy; 3 Santa Lucia Foundation, Rome, Italy

Dear Sirs,

We aimed to verify whether transient global amnesia (TGA) could be sustained by acute changes of amino acids in the blood, analogous to the transient memory loss induced by experimental tryptophan depletion [1, 2].

Plasma amino acids were studied in control healthy subjects (aged 48–65) and in patients (aged 49–70) during the amnesic attack (*n* = 9) and longitudinally at 3 days (*n* = 12) and 16–24 months (*n* = 9) after the amnesic episode, or retrospectively in patients (*n* = 9) who had an amnesic episode 2–3 years before. Routine blood tests for metabolic disorders were also studied. Patients who fulfilled the operational criteria for TGA showed massive impairment of both retrograde and anterograde episodic memory during the acute phase [3–5], but 3–4 days later the memory tests were within the normal range in every case [6]. Brain RMN and EEG examinations showed no relevant abnormalities, even though minimal theta discharges and/or chronic vascular encephalopathy were observed in about half of the patients [5]. Emotional stress and intense physical exertion were reported by 9/21 patients [7]. The study has been approved by the appropriate ethics committee and has therefore been performed in accordance with the ethical standards laid down in the 1964 Declaration of Helsinki and its later amendments. All persons gave their informed consent prior to their inclusion in the study.

**Fig. 1:**
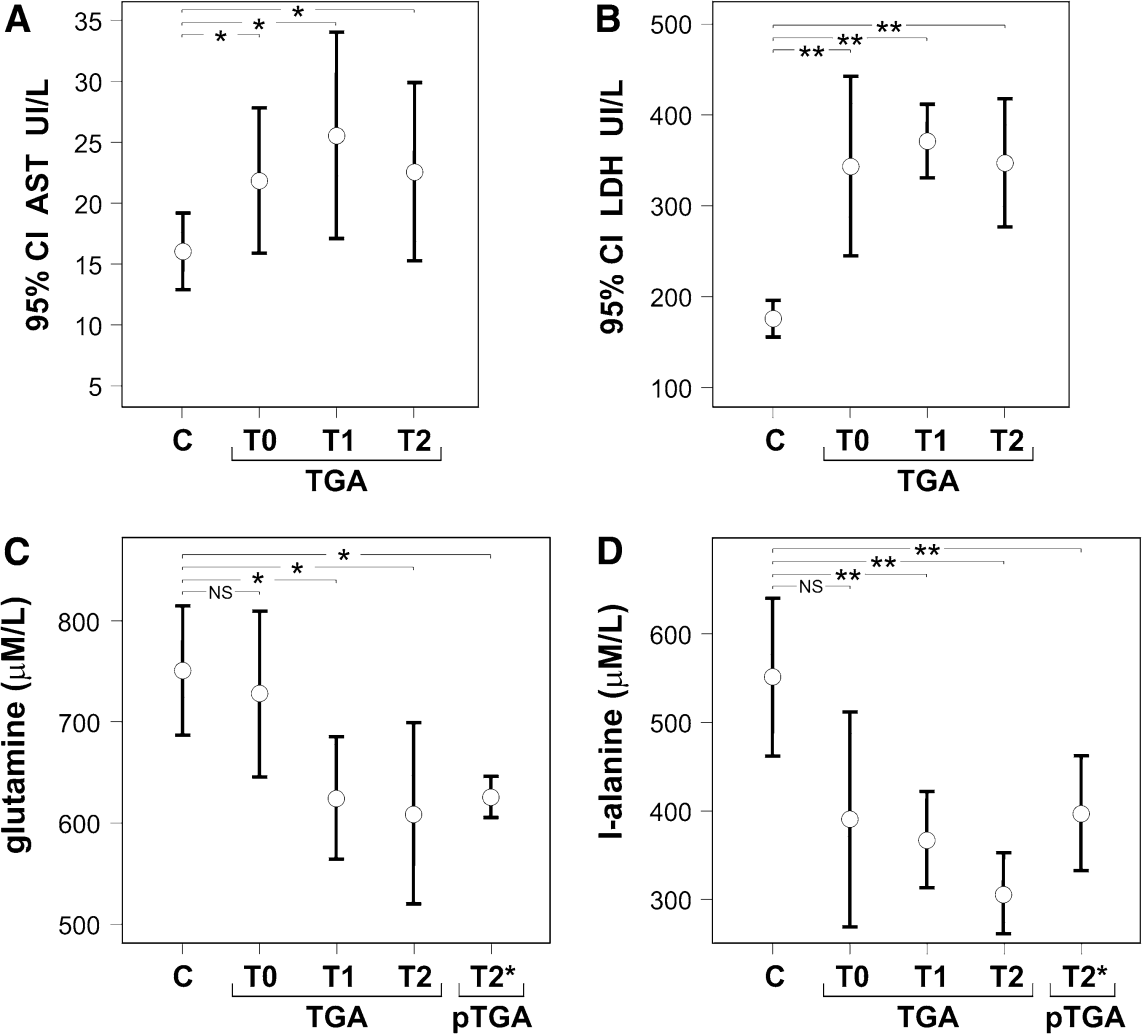
**a** total activities (UI/L) of aspartate aminotransferase (AST), and **b** lactate dehydrogenase (LDH) in blood of normal subjects (*n* = 9) and patients suffering from an episode of transient global amnesia (TGA), studied during the amnesic attack (TGA-T0) (*n* = 9), and at 3 days (TGA-T1) (*n* = 12), and 16–24 months (TGA-T2) (*n* = 9) after the amnesic episode, **c** concentrations (mean ± SD, μM/L) of free amino acid glutamine, and **d** l-alanine in plasma of normal subjects (*n* = 9) and patients suffering from an episode of transient global amnesia, studied either prospectively during the amnesic attack (TGA-T0) (*n* = 9), and at 3 days (TGA-T1) (*n* = 12), and 16–24 months (TGA-T2) (*n* = 9) after the amnesic episode, or retrospectively in a second group of patients that had experienced a previous amnesic episode (pTGA-T2*) 2–3 years before (*n* = 9). One-way ANOVA followed by Bonferroni’s test, n.s. nonsignificant, *p < 0.05, **p < 0.01

Blood tests were free from abnormalities, but lactate dehydrogenase (LDH) and aspartate aminotransferase (AST), respectively involved in anaerobic glycolysis and in amino acid metabolism, came out to be constantly overexpressed during, as well as shortly and long after, the amnesic attack (Fig. 1). The levels of plasma amino acids were similar in controls and in patients with a current TGA attack (Table 1). However, the values of glutamine and l-alanine, but not of the other amino acids, were significantly lower in patients at 3 days (T1) and at 16–24 months (T2) from the amnesic episode compared to age-matched healthy control subjects (Fig. 1). Furthermore, levels of glutamine (*p* < 0.05) and l-alanine (*p* < 0.01) were significantly lower in patients recruited for the study of plasma amino acids 2 to 3 years after a previous TGA episode, compared to controls (Fig. 1). Note that the significant difference between control subjects and patients with a previous TGA episode is enhanced by associating the co-occurrence of low levels of glutamine and l-alanine in the latter group (*p* < 0.0001). To evaluate how the small sample sizes influence the results in our study we performed power analysis. Analysis of group sample sizes of nine for values of glutamine of control healthy subjects and of patients with a previous TGA episode achieved 90 % power to detect a difference of 142.0 between the null hypothesis that both group means are 751.0 and the alternative hypothesis that the mean of TGA group is 609.0 with known group standard deviations of 82.0 and 107.0, respectively, and with a significance level (alpha) of 0.05, using a *t* test and assuming that the actual distribution is normal. However, the apparent increase of glutamine and l-alanine during a TGA attack was highly variable, thus the differences from T0 to T1–T2 were not significant within the TGA group (Fig. 1). Such non-significant increase of glutamine and l-alanine had not been influenced by an undetermined food intake prior of the amnesic attack, since the values of 1-methylhistidine and 3-methylhistidine, considered an index of daily protein ingestion [8], were similar in the blood samples obtained at the time of the amnesic attack and long after the amnesic episode in the same patients (Table 1). These data suggest that the nonsignificant increase of glutamine and l-alanine during the amnesic attack should be linked to the variable duration of the transient amnesic attack, which may last from 15 min to 24 h. [4].

**Table 1:** Concentrations of free plasma amino acids

Amino acid	Control subjects	T0-TGA	T1-TGA	T2-TGA	T2*-pTGA	*p*
b-alanine	9.536 ± 4.062	7.189 ± 3.234	10.380 ± 4.646	10.000 ± 0.756	11.182 ± 5.820	n.s.
l-alanine	540.22 ± 103.48	419.43 ± 106.36	368.00 ± 86.02	307.38 ± 54.74	397.44 ± 84.34	<0.01
Glutamine	751.33 ± 82.93	727.86 ± 88.78	625.08 ± 95.17	609.50 ± 107.23	625.89 ± 26.38	<0.05
Isoleucine	84.04 ± 23.85	82.57 ± 26.77	78.08 ± 21.27	74.25 ± 14.69	72.43 ± 22.71	n.s.
Leucine	141.40 ± 44.69	149.46 ± 46.05	145.83 ± 29.14	138.88 ± 18.92	131.92 ± 42.75	n.s.
1-methylhistidine	7.00 ± 9.90	5.32 ± 4.76	5.60 ± 4.55	7.00 ± 6.85	9.29 ± 11.87	n.s.
3-methylhistidine	7.87 ± 8.16	5.97 ± 2.49	4.93 ± 2.80	3.63 ± 1.92	5.11 ± 1.11	n.s.
Methionine	28.91 ± 7.32	25.46 ± 4.77	26.83 ± 5.00	34.00 ± 5.21	26.03 ± 4.24	n.s.
Phenylalanine	69.17 ± 15.46	69.69 ± 13.35	72.66 ± 31.51	64.75 ± 12.59	63.20 ± 10.88	n.s.
Sarcosine	5.96 ± 1.56	4.19 ± 1.95	5.71 ± 3.63	4.38 ± 0.91	4.54 ± 0.90	n.s.
Tryptophan	50.47 ± 8.22	45.63 ± 8.76	44.14 ± 12.11	44.25 ± 7.96	49.16 ± 10.7	n.s.
Tyrosine	74.50 ± 26.11	70.89 ± 13.86	68.41 ± 6.36	71.00 ± 9.27	73.81 ± 27.54	n.s.
Valine	266.44 ± 35.76	254.71 ± 77.88	257.92 ± 41.82	250.63 ± 26.91	241.44 ± 66.21	n.s.
Tryptophan/LNAA ratio	0.078 ± 0.017	0.073 ± 0.017	0.069 ± 0.018	0.070 ± 0.010	0.0829 ± 0.018	n.s.

Concentrations (μM/L) of free amino acids (mean ± SD) in plasma of normal control subjects (*n* = 9) and of patients with transient global amnesia (TGA) (*n* = 21). Patients with TGA were studied either prospectively during the amnesic attack (T0-TGA) (*n* = 9), at 3 days (T1-TGA) (*n* = 12), and at 16–24 months (T2-TGA) *(n* = 9) after the amnesic episode, or retrospectively in a second group of patients (*n* = 9) that had suffered a previous amnesic attack (T2*-pTGA) 2–3 years before

Comparisons between groups and within groups *n.s.* nonsignificant, *p < 0.05, **p < 0.01 glutamine and l-alanine control subjects versus T1-TGA, T2-TGA, T2*-pTGA

How can the low steady state of glutamine and l-alanine be interconnected to the overexpression of AST and LDH detected in patients after TGA? LDH catalyzes the interconversion of pyruvate to lactate during anaerobic glycolysis in skeletal muscle, while AST catalyzes the interconversion of aspartate and α-ketoglutarate to glutamate and oxaloacetate. In turn, glutamate, through the action of alanine aminotransferase, can transfer its amino group to pyruvate, a product of muscle glycolysis, forming l-alanine; moreover, glutamine is synthesized by the enzyme glutamine synthetase, adding a second amino group to glutamate [9, 10]. Glutamate and glutamine are the most abundant amino acids in the brain: the CSF/plasma ratio for glutamine is indeed more than 0.90, whereas the concentrations of all other amino acids in CSF are ~10 % or less than the plasma concentrations [11, 12]. An undue increase of glutamine and l-alanine in the blood may impair their clearance from the brain and the homeostasis of glutamate precipitating the amnesic attack. It is worth noting that the liberation of excitotoxic neurotransmitters in the brain, specifically glutamate, has been suggested as the pathogenic mechanism of TGA [13]. Moreover, MR spectroscopy demonstrated a lactate peak in the hippocampus of patients with TGA, indicating an acute metabolic stress of CA-1 neurons in these patients [14]. In conclusion, although TGA is by definition a transient and reversible clinical phenomenon lasting no more than 24 h, patients who have undergone a previous TGA episode are characterized by permanent low levels of glutamine and l-alanine and by high levels of AST and LDH in blood, which probably represent constitutive metabolic traits distinctive from that of control subjects. Further research has to be done to evaluate whether such metabolic factors may occasionally predispose to unbalanced catabolism of amino acids and to high levels of glutamate, sustaining a TGA attack.
